# Autophagy-dependent survival is controlled with a unique regulatory network upon various cellular stress events

**DOI:** 10.1038/s41419-021-03599-7

**Published:** 2021-03-23

**Authors:** Orsolya Kapuy, Marianna Holczer, Margita Márton, Tamás Korcsmáros

**Affiliations:** 1 Institute of Biochemistry and Molecular Biology, Semmelweis University, Budapest, Hungary; 2 Earlham Institute, Norwich, UK; 3 Quadram Institute Bioscience, Norwich, UK

**Keywords:** Biochemistry, Molecular biology

## Abstract

Although autophagy is a type of programmed cell death, it is also essential for cell survival upon tolerable level of various stress events. For the cell to respond adequately to an external and/or internal stimulus induced by cellular stress, autophagy must be controlled in a highly regulated manner. By using systems biology techniques, here we explore the dynamical features of autophagy induction. We propose that the switch-like characteristic of autophagy induction is achieved by a control network, containing essential feedback loops of four components, so-called autophagy inducer, autophagy controller, mTORC1 and autophagy executor, respectively. We show how an autophagy inducer is capable to turn on autophagy in a cellular stress-specific way. The autophagy controller acts as a molecular switch and not only promotes autophagy but also blocks the permanent hyperactivation of the process via downregulating the autophagy inducer. In this theoretical analysis, we explore in detail the properties of all four proposed controlling elements and their connections. Here we also prove that the kinetic features of this control network can be considered accurate in various stress processes (such as starvation, endoplasmic reticulum stress and oxidative stress), even if the exact components may be different. The robust response of the resulting control network is essential during cellular stress.

## Introduction

The Greek word ‘autophagy’ means ‘self-eating’, referring to the ability of the cells to digest their own components with respect to various external and internal signals. Basal autophagy is observed even under physiological conditions; however, this process gets more efficient upon increasing cellular stress level^1–3^. Traditionally, autophagy was classified as a cell death mechanism^4^; however, many scientific results have been revealed that autophagy also has an essential role in cellular survival upon various stress events (such as starvation or endoplasmic reticulum (ER) stress)^5,6^. These data clearly suggest that the crucial function of autophagy is to maintain cellular homoeostasis, while excessive level of permanent autophagy can result in cell death^5,7–9^.

Due to the essential role of autophagy in regulating cellular homoeostasis and stress response, both the induction and the downregulation of the process are tightly controlled^10^. One of the most important elements of the process is unc51-like autophagy activating kinase 1/2 (ULK1/2), the mammalian homologue of yeast Atg1^11,12^. ULK1/2 controls the early stage of autophagy via forming a so-called autophagy induction complex with ATG13, ATG101 and FIP200^13–16^. This complex can phosphorylate Beclin1, the mammalian homologue of yeast Atg6^17^. Beclin1 forms a multiprotein complex with other molecules (such as VPS34, ATG14 and AMBRA1), to enhance the formation of the double-membrane structure (so-called isolation membrane) to engulf cytoplasmic material for autophagosome formation^10,18^. Although the molecular mechanism of autophagy induction seems to be universal, the process also has stress-specific regulators upon various stress events (i.e., starvation, oxidative exposure, and ER stress).

Aminoacid- or glucose-deprivation-induced cellular stress is tightly controlled by both mammalian target of rapamycin (mTOR) and AMP-protein kinase (AMPK)^19–21^. mTOR, when in a complex with other proteins (such as Raptor, MLST8, PRAS40 and Deptor), called mTORC1 is the master regulator of cellular growth and metabolism^22^. AMPK is a heterotrimeric protein complex and it has an essential role in maintaining energy homoeostasis by sensing the change of cellular AMP/ATP ratio^19^. mTORC1 inhibits autophagy under nutrient-rich conditions, meanwhile AMPK promotes the autophagy upon starvation^23^. The precise crosstalk between mTORC1 and AMPK is achieved via a double-negative feedback loop^24,25^. In addition, both kinases regulate ULK1/2 directly. mTORC1-dependent phosphorylation of ULK1/2 results in its inactivation, whereas AMPK is able to induce ULK1/2^23,26^. Interestingly, ULK1/2 kinase inhibits both AMPK and mTORC1 via phosphorylation, generating negative and double-negative feedback loops in the control network^23,27–30^.

The nuclear factor erythroid 2-related factor 2 (NRF2) has a key role to enable cell adaptation to oxidative stress by promoting the transcription of more than 2000, mainly cytoprotective genes^31–33^. NRF2 is bound to KEAP1 into an inactive complex under physiological conditions; however, p62 (also known as SQSMT1; sequestosome) quickly gets activated upon oxidative stress^34,35^. Active p62 has a high binding affinity to KEAP1, therefore enhancing the dissociation of active NRF2 from KEAP1^36–38^. Besides, p62 targets proteins to be transferred to autophagosome and induces their autophagy-dependent degradation^34,39^. Moreover, NRF2 promotes the expression of many autophagy genes, such as *ATG3*, *ATG5*, *ATG7*, *p62* and *GABARAPL1* upon oxidative stress^40^. Recently, we have also shown the regulatory connection between AMPK and NRF2 upon oxidative stress^41^. Although AMPK has a transient activation followed by NRF2 induction during oxidative stress, we found that NRF2 deficiency resulted in a permanent activation of AMPK. Our results show that NRF2 is essential to downregulate autophagy via repressing *AMPK* transcription upon prolonged oxidative stress^41^.

Interestingly, ER stress induced by harmful external and internal effects (such as oxidative agents, accumulation of not properly folded proteins) also immediately induces the formation of autophagosomes^42^. The ER stress response mechanism turns on a complex network of signalling pathways, called unfolded protein response (UPR). UPR has three well-defined branches controlled by ER membrane-associated proteins, called IRE1 (inositol requiring 1), PERK (PKR-like ER kinase) and ATF6 (activating transcription factor 6)^43^. Although both IRE1 and ATF6 mainly induce UPR target genes (such as chaperones), the key role of PERK pathway is to block the protein translation^43^. According to the level of ER stress, each branches of UPR are able to enhance autophagy^44^. It has shown that tolerable ER stress results in autophagy induction to promote cellular survival, but excessive level of ER stress leads to transient autophagy followed by apoptotic cell death^45^. With systems biology methods, we have also claimed that the feedback loops between the branches of UPR are crucial to the proper cellular life-and-death decision upon ER stress^46,47^.

Although many biologists are focusing on the mechanism of autophagy induced by various cellular stress events, the dynamical features of the regulatory network of cellular stress-specific response mechanism have not been explored yet. By using systems biology techniques, here we present a general model of autophagy induction by focusing on the key elements and feedback loops. To give a qualitative description about the control network, we studied both the induction and the downregulation of autophagy upon cellular stress. This approach is able to analyse the dynamical characteristic of the control network; therefore, it can result in medically relevant observations and results (e.g., disease-specific drug targets or biomarkers for autophagy malfunction).

## Materials and methods

### A control network with feedback loops can describe a dynamic autophagy regulation system

Mathematical models are useful to understand the precise molecular mechanisms that control important aspects of cell physiology, such as cell growth and division or cellular life-and-death decision^48,49^. The theoretical modelling of a biological system can give a proper directionality to molecular biological experiments by giving a qualitative description about the dynamical characteristic of the cellular regulatory networks.

**Fig. 1:**
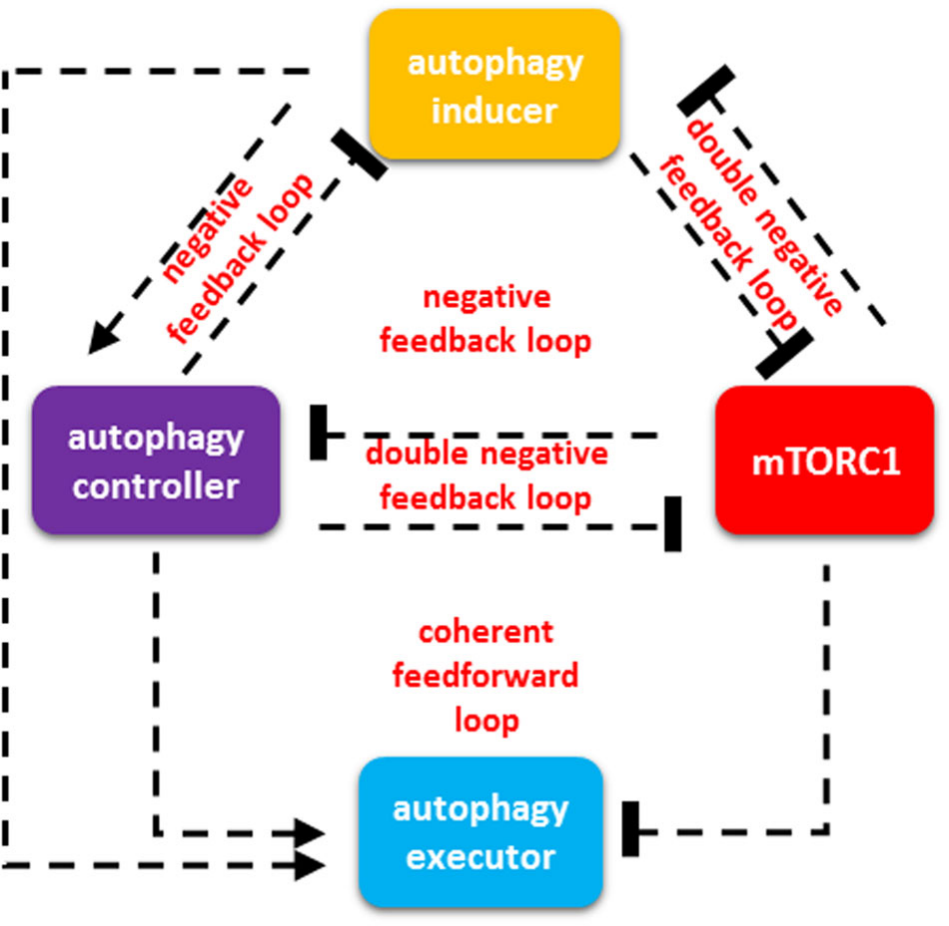
The comprehensive wiring diagram of autophagy induction upon cellular stress. A general wiring diagram to describe the precise mechanism of autophagy induction. The regulatory elements and their connections of life-and-death decision when the autophagy inducers (AUIN), the autophagy controllers (AUCO), the autophagy executors (AUEX) and the elements of mTORC1 pathway are grouped together in isolated orange, purple, blue and red boxes, respectively. Dashed lines show how the molecules can influence each other. Blocked end lines denote inhibition.

Our analysis mainly focuses on the kinetic features of autophagy induction upon various cellular stress events, such as starvation, ER stress or oxidative exposure. We have thoroughly studied more than 100 scientific papers from the life science and medical fields to build a control network model of autophagy-dependent survival. For the references of the publications used to build the model, see Supplementary Table 1. According to the already published scientific data, we propose that regulated autophagy induction can be described by a wiring diagram of four different regulators, called autophagy inducer (AUIN), autophagy controller (AUCO), mTORC1 and autophagy executors (AUEX), respectively (Fig. 1). The biological evidence suggests that AUIN is able to promote autophagy via indirect upregulation of AUEX by both upregulating AUCO and downregulating mTORC1. Corresponding to the already published data, we claim that mTORC1 prevents the induction of AUIN and AUCO. The common features of AUCO are as follows: (1) inhibits mTORC1, (2) inhibits AUIN and (3) promotes AUEX. Based on experimental data, we propose when AUEX is active, the autophagy-dependent cell survival is turned on. As AUCO can also inhibit both mTORC1 and AUIN, two negative feedback loops (i.e., AUCO ┤ AUIN − > AUCO and AUCO ┤ mTORC1 ┤ AUIN − > AUCO) are generated. Both AUIN and AUCO promote AUEX referring to their importance in autophagy induction.

Each component has an active and an inactive form in the model. The cellular stress is used as an input parameter in the control network. For details about the codes and software used for simulations, see the Supplementary Information.

The question immediately arises, which components of the control network are named as AUIN, AUCO and AUEX. Based on data obtained from the literature, we created a table containing the potential regulators of autophagy induction. With the already published scientific data, we propose which components of autophagy regulation could be AUIN, AUCO and AUCO, and details about them are collected in Supplementary Table 1. For example, AMPK and GADD34 act like AUIN; NRF2, CHOP and ULK1/2 are considered to be AUCO; whereas ATG5, ATG7 and Beclin1 can work as AUEX. We propose that this control network can be regulated in a stress-type-specific manner. The three columns of the Supplementary Table 1 contain all the possible AUIN, AUCO and AUEX at mTOR inhibition, upon ER stress or during oxidative stress. Besides, all the experimentally proved regulators of the network diagram with the possible regulatory connections are shown in Supplementary Fig. 1. All the relevant literature about the sign of the regulatory connections (i.e., the control molecules activate or inhibit eachother) are collected in Supplementary Table 2.

## Results

### The proper behaviour of the model is proved upon three various stress events

To confirm the accuracy of our autophagy regulation model, here we investigate the kinetic features of stress-type-specific autophagy controlling networks. To support our observations with relevant and existing biological evidence, we analyse here three, well-defined stress events as follows: starvation, ER stress and oxidative exposure.

#### Upon starvation stress

**Fig. 2:**
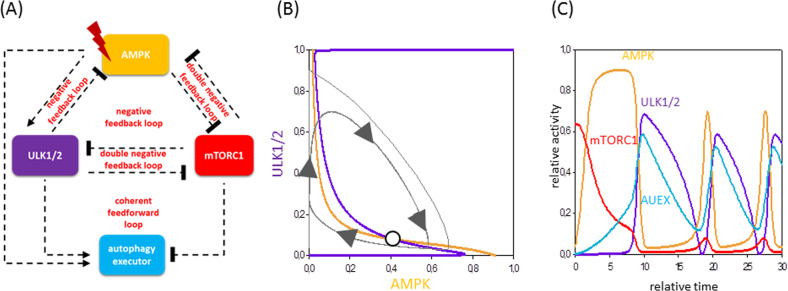
System-level feedbacks guarantee a robust stress response mechanism upon starvation. **A** The wiring diagram is plotted to describe the precise mechanism of autophagy induction under starvation. Dashed lines show how the molecules can influence each other. Blocked end lines denote inhibition. **B** Phase plane diagrams are plotted upon excessive levels of stress. The balance curves of AMPK (orange) and ULK1/2 (purple) are plotted. The phase plane is shown for stress = 0.75. Intersection of nullclines represents the unstable (unfilled circle) steady state. Trajectories are depicted with grey lines. The temporal dynamics (**C**) is simulated with stress = 0.75. For details about the codes and software used for simulations, see the Supplementary Information.

Biologists have already shown that cellular food supply is controlled by the AMPK-ULK1/2-mTORC1 regulatory triangle^23^. AMPK is able to promote autophagy (i.e., increasing the relative activity of ATG genes) by phosphorylating ULK1/2, the key regulator of autophagosome formation^23,50^. Besides, AMPK directly inhibits mTORC1 via phosphorylation upon nutrient depletion^23,51–53^. mTORC1 inhibits autophagy under nutrient-rich condition by downregulating both ULK1/2 and AMPK^23–26^. Interestingly, ULK1/2 inhibits AMPK, generating a negative feedback loop in the control network of autophagy induction^27,54^ (Fig. 2A).

To study the role of experimentally proved regulatory motifs in the control network, the overall steady-state response of the system is computed, generating a so-called phase plane diagram. In this case, our ordinary differential equation system is simplified to a pair of differential equation for ULK1/2/dt and AMPK/dt, respectively. We assume that all the other components are in steady state. The coordinate system is spanned by ULK1/2 and AMPK, and then the so-called balance curves, namely ULK1/2/dt = 0 (purple) and AMPK/dt = 0 (orange) are plotted (Fig. 2B). Balance curve (or mathematically called as nullcline) means that the rate of activation of the given component is exactly balanced by the rate of its degradation. The nullcline of ULK1/2 is S-shaped due to the double-negative feedback loops in the control network. Where the nullclines intersect each other upon starvation stress, the control network has one unstable steady state.

In case of starvation, the negative feedback loop between ULK1/2 and AMPK results in a sustained oscillatory characteristic (Fig. 2B, C). AMPK ensures the activation of ULK1/2, which, after a certain time delay, promotes the inactivation of AMPK (Fig. 2B). The phase plane diagram of ULK1/2 and AMPK with one unstable intersection shows a limit cycle oscillation, where grey arrows indicate the direction of motion among the limit cycle. Corresponding to our previous experimental results^55^, time course of sustained oscillation of ULK1/2 and AMPK has also depicted and AUEX gets periodically active (Fig. 2C) too, suggesting that autophagy has an ON and OFF characteristic under starvation.

#### Upon ER stress

**Fig. 3:**
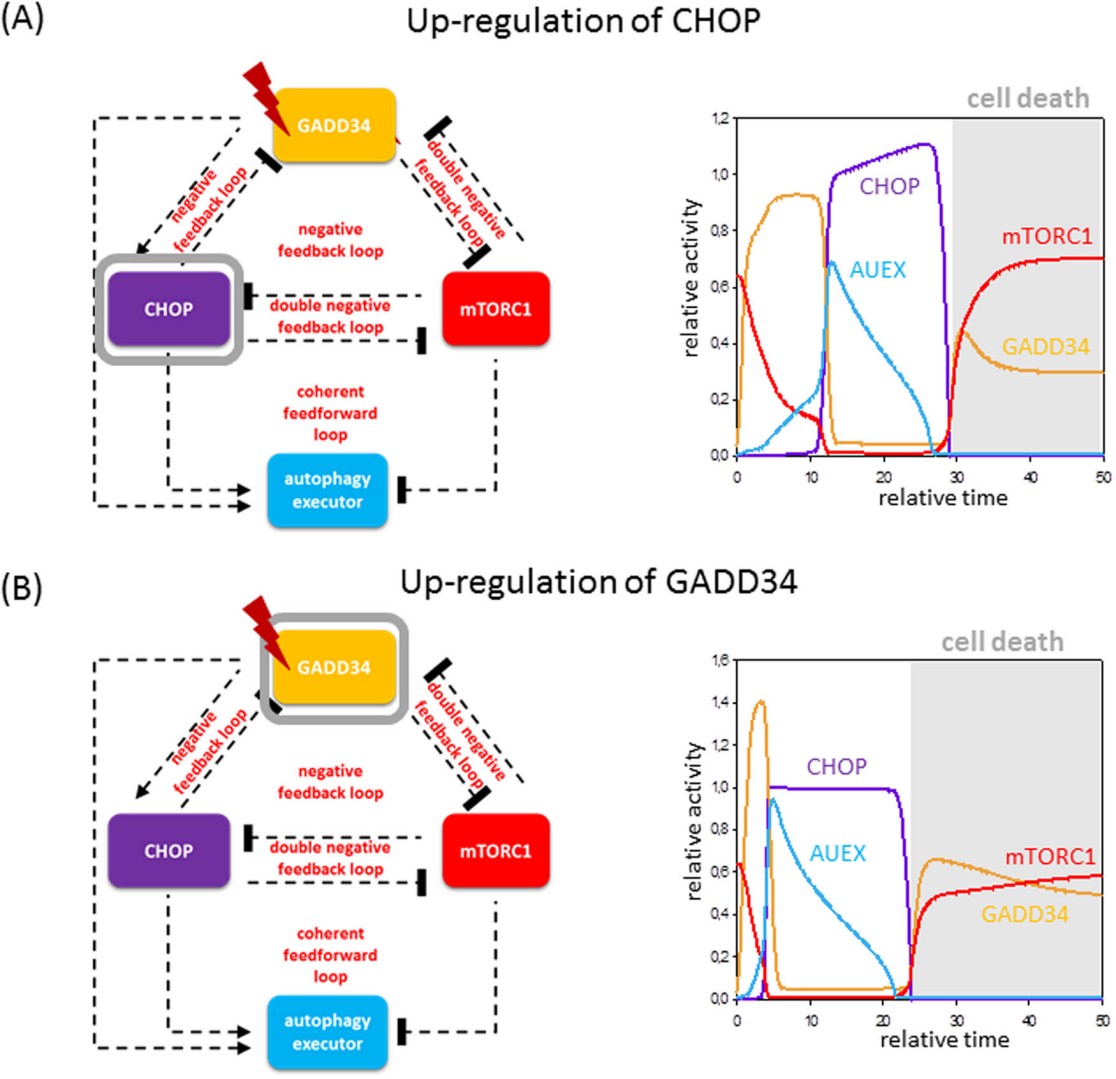
Both GADD34 and CHOP have important functions at endoplasmic reticulum stress: upregulation of CHOP or GADD34. The computational simulations are determined in the overexpression of **A** CHOP and **B** GADD34 upon excessive levels of cellular stress. The temporal dynamics is simulated with high stress (stress = 9) combined with **A** CHOP-T = 1.25 or **B** GADD34-T = 2. Grey background refers to possible cell death. For details about the codes and software used for simulations, see the Supplementary Information.

**Fig. 4:**
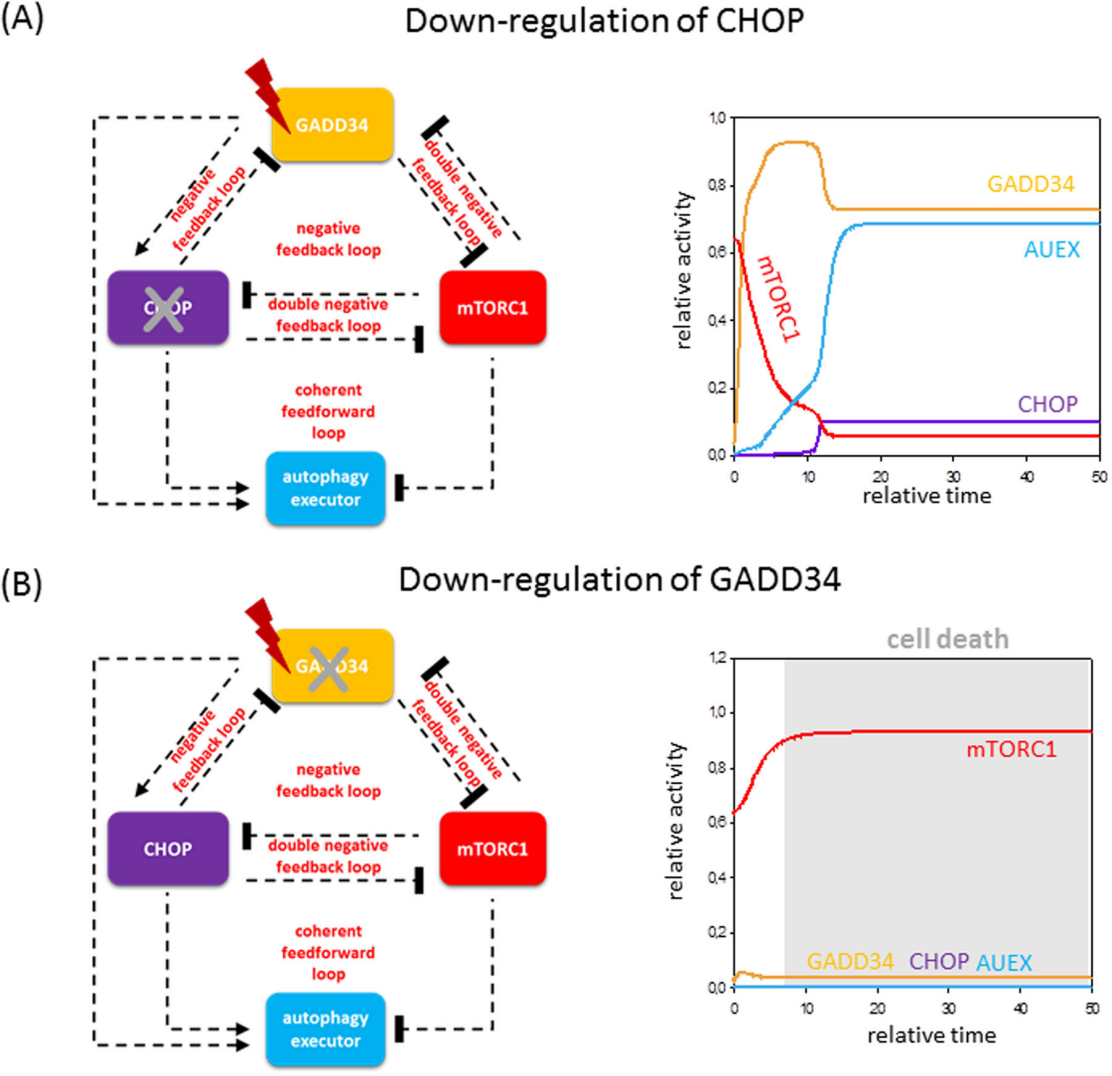
Both GADD34 and CHOP have important functions at endoplasmic reticulum stress: downregulation of CHOP or GADD34. The computational simulations are determined in the absence of **A** CHOP and **B** GADD34 upon excessive levels of cellular stress. The temporal dynamics is simulated with high stress (stress = 9) combined with **A** CHOP-T = 0.1 or **B** GADD34-T = 0.1. Grey background refers to possible cell death. For details about the codes and software used for simulations, see the Supplementary Information.

It has been already experimentally confirmed that autophagy-dependent survival is followed by apoptotic cell death upon excessive level of ER stress and this mechanism is under the control of UPR^5,6,45^. The biological evidence suppose that two signal transducers of UPR, namely GADD34 and CHOP, have essential roles in ER stress response^56,57^. According to the experimental data, GADD34 promotes autophagy upon ER stress via downregulating mTORC1^58^. CHOP is a transcription factor that controls gene transcription involved in apoptosis^59^, but it also has a positive effect on transcription of various autophagy genes (*p62*, *ATG3* and *ATG12*)^60^. Here we suggest that GADD34 acts like AUIN, whereas CHOP might be AUCO with respect to ER stress. To confirm our assumption, we compare several well-known experimental data about GADD34 and CHOP to the kinetic analysis of our control network (Figs. 3 and 4).

Upregulation of either CHOP or GADD34 results in a short and transient activation of autophagy followed by the quick re-activation of mTORC1 and a possible cell death (Fig. 3A, B). These results nicely refer to that experimental data when overexpression of either CHOP or GADD34 rapidly turns on apoptotic cell death^59,61^.

Besides, it has experimentally already shown that addition of catalytically inactive GADD34 (GADD34ΔC/ΔC) result in pre-mature cell death in the presence of ER stress^62^. It is also well-known that CHOP-deleted cells are much less sensitive to ER stress compared to wild-type strain^63^. Consistent with the experimental data, downregulation of CHOP causes hyperactivation of both GADD34 and AUEX (Fig. 4A). In contrast, depletion of GADD34 completely diminishes autophagy, i.e., neither CHOP nor AUEX gets activated in our time-course simulation (Fig. 4B).

Our analysis clearly points out that GADD34 belongs more likely to AUIN, whereas CHOP might carry the dynamical characteristic of AUCO, further confirming that our general model can be properly used to describe ER stress response mechanism.

#### Upon oxidative stress

**Fig. 5:**
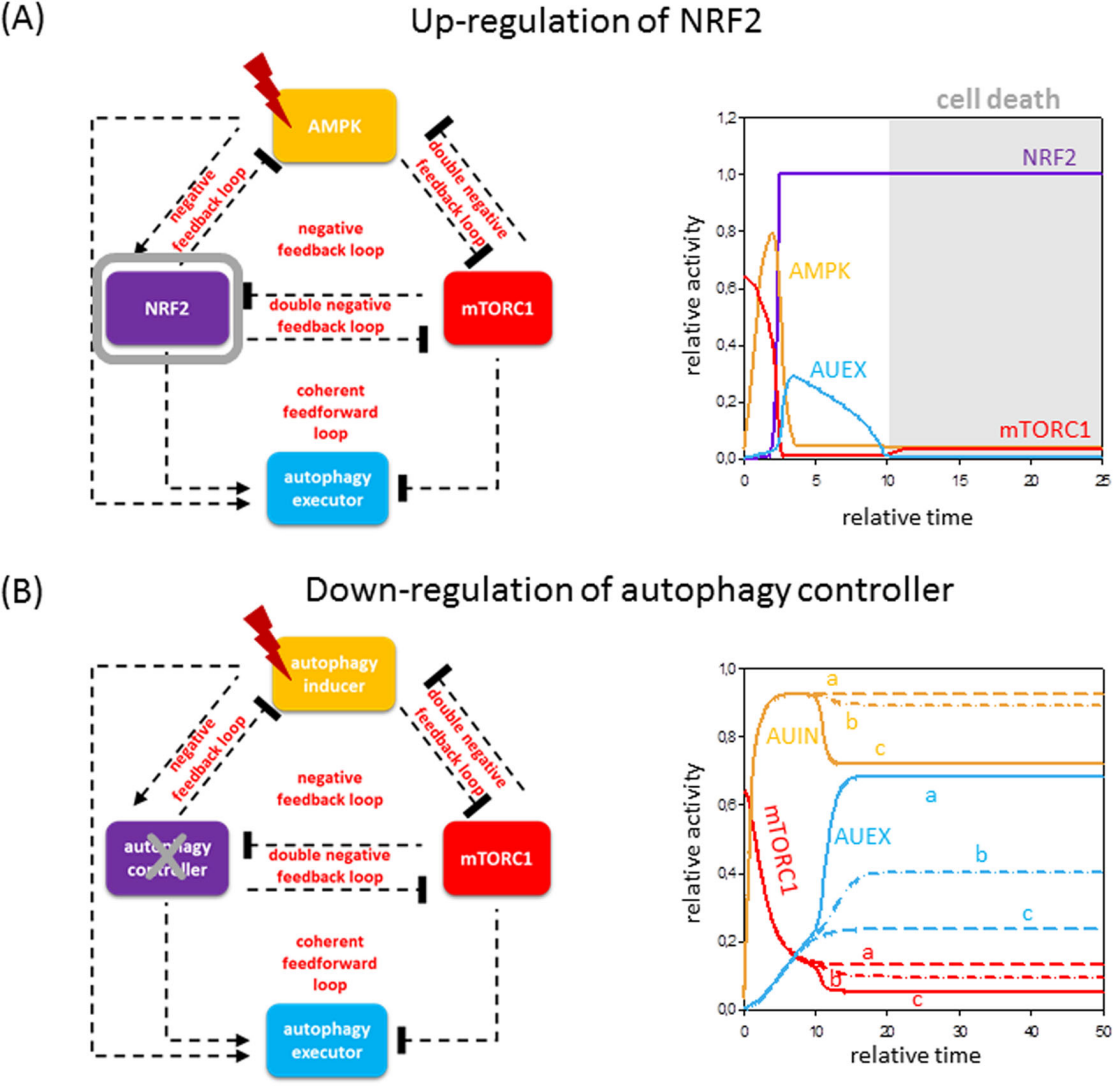
The precise level of autophagy controller is essential in stress response. Different mutant phenotypes are simulated: **A** Keap1 depletion (NRF2 activation) is mimicked upon excessive level of cellular stress (stress = 7.5, kaac = 5). **B** Depletion of autophagy controller is achieved on various levels (stress = 7.5, AUCO-T = 0.01, see lines marked with ‘a’; stress = 0.03, see lines marked with ‘b’; stress = 0.05, see lines marked with ‘c’). Grey background refers to possible cell death. For details about the codes and software used for simulations, see the Supplementary Information.

In our previous biological study, we have shown that NRF2 has an essential role in downregulating *AMPK* upon oxidative stress^41^, suggesting NRF2 works as AUCO, whereas other data assume that AMPK might be AUIN^21^ in our model (Fig. 5A). Similar to starvation, upon oxidative exposure, AMPK is essential to induce autophagy, but alone is not sufficient to maintain autophagy. AMPK turns on NRF2, which later inhibits AMPK via a negative feedback loop.

Gonzales et al.^64^ has recently shown experimentally that depletion of KEAP1 results in a downregulation of autophagy upon oxidative stress, but the molecular mechanism was not confirmed. As the key role of KEAP1 is to keep NRF2 in an inactive complex, we mimic KEAP1 depletion by increasing the amount of NRF2 (Fig. 5A). Although NRF2 has a positive effect on autophagy, its high level immediately inhibits the activation of AMPK. AMPK is essential for the induction of autophagy; therefore, autophagy remains inactive and cells might enhance cell death.

These results clearly show that NRF2 is the key controller of autophagy by switching ON and OFF the process upon oxidative stress.

### AUCOs are the main switch elements for autophagy regulation upon cellular stress

Our analysis suggests that AUIN is essential for autophagy induction, but the exact role of AUCO is still a bit vague, as both theoretical and biological data have shown that the inhibition of various AUCOs (such as ULK1/2, CHOP or NRF2) result in various response. Therefore, we study the dynamical characteristic of autophagy induction when the total level of AUCO is systematically decreased upon cellular stress (Fig. 5B).

In that case, when the amount of AUCO is reduced by not so heavily (AUCO-T = 0.03 or 0.05, stress = 7.5), AUIN together with the reduced amount of AUCO is able to enhance AUEX (see lines ‘a’ and ‘b’ on Fig. 5B). As AUCO is not strong enough to downregulate AUIN properly, therefore autophagy gets hyperactivated upon excessive level of cellular stress. This kinetic behaviour was experimentally observed when the relative activity of NRF2 was depleted^41^, whereas cellular survival was significantly increased in the absence of CHOP^63,65^.

When AUCO-T is fully inhibited (AUCO-T = 0.01, stress = 7.5), AUEX cannot be active (see lines ‘c’ on Fig. 5B). Although AUIN becomes high and tries to induce AUEX (and AUCO as well), it alone is not sufficient to maintain autophagy. However, the high level of AUIN is sufficient to keep mTORC1 inactive. This dynamical feature is completely consistent with that experimental data when ULK1/2 is inhibited during starvation^24^.

Our analysis shows that a proper level of AUCO is essential for both turning ON and OFF autophagy, acting like a switch upon cellular stress.

## Discussion and conclusions

Inspired by both experimental data and our previous theoretical analysis, we have created a general control model of autophagy regulation to analyse the potential roles of elements and feedback loops describing the dynamical characteristic of the response mechanism upon various cellular stress events. In this work, we have explored the systems-level properties of the control network using a mathematical model.

It is well-known that mTORC1 level is high and inhibits autophagy at physiological conditions. Here we grouped the molecules controlling autophagy regulation into three groups, called as AUIN, AUCO and AUEX, respectively (Fig. 1). The double-negative feedback loops between AUIN and AUCO; AUCO and mTORC1 generate bistability in the system with one physiological state and one autophagy state. Interestingly, two negative feedback loops are also present between AUIN and AUCO, and between AUCO and mTORC1 in the control network, suggesting that AUIN-AUCO-mTORC1 regulatory triangle has a critical effect on stress response mechanism. The possible regulatory connections supported by biological evidence are collected in Supplementary Table 2.

The question immediately arises, who are the exact elements hiding under our group names (i.e., AUIN, AUCO and AUEX) with respect to various cellular stress mechanisms. The presence of some proteins of the control network has been already experimentally proved in case of rapamycin treatment or starvation, revealing that AUIN might be AMPK and AUCO might be ULK1/2 (Fig. 2); however, these elements are not properly studied upon ER stress. Here we confirm that GADD34 and CHOP are well-known regulators of autophagy upon ER stress (Figs. 3 and 4), but these results need further experimental clarification in the future (the possible regulators are collected in Supplementary Table 1 and Supplementary Fig. 1). We also claim that always more than one protein from the same group takes part in the reaction to guarantee a robust stress response mechanism in any circumstances.

Although NRF2, CHOP and ULK1/2 are all called AUCO in our model, diminishing one of them results in different outcomes upon cellular stress. Similar to the published experimental data, downregulation of ULK1/2 completely blocks proper autophagy^13,23,24,66^, whereas either CHOP or NRF2 depletion results in the hyperactivation of the autophagy^41,63,65^ (Fig. 5B). In each case, AUIN (AMPK or GADD34) gets activated leading to the downregulation of mTORC1. AUIN promotes the activation of both AUCO and AUEX. As AUCO is essential for AUEX induction, our computer simulations confirm that in the total absence of AUCO, AUEX cannot be active. Meanwhile hyperactivation of AUEX can only be observed in that case if AUCO is not fully inactivated, assuming that neither NRF2 nor CHOP depletion results in complete inactivation of AUCO in the control network. These analyses also suggest that besides the cellular stress-specific AUCOs (i.e., NRF2 in oxidative stress and CHOP in ER stress), a non-stress-specific AUCO might be always present upon cellular stress and has an important role in regulating autophagy. We propose that this non-stress-specific AUCO is ULK1/2, as it is essential for autophagosome formation and is completely sufficient to block autophagy during starvation. However, these connections have to be clarified later experimentally.

Our computational model of the control network suggests that AUCO is the key switch controlling the jump between ON and OFF state of autophagy induction with respect to cellular stress level. Corresponding to our systems biological analysis, we propose that ULK1/2 is the main switch, whereas stress-specific side switches are also operating to make a precise answer upon cellular stress. For example, NRF2 could be an oxidative stress-dependent side switch. If this theory is valid, the main and side switches have to crosstalk to each other to generate an accurate cellular decision. To further clarify this assumption, we checked the possible connections between NRF2 and ULK1/2. As ULK1/2 is a kinase, first we identified potential Ser and Thr phosphorylation sites on NRF2 with Group-based Prediction System 5.0^67^ and NetPhos 3.1^68^. We found more than one consensus phosphorylation motifs of ULK1/2 on NRF2, suggesting that ULK1/2 might be able to control the NRF2 activity. By using the online available NRF2ome^69^, we also found that NRF2 is able to bind the promoter region of ULK2, suggesting that NRF2 might be a potential transcription factor of the kinase. It has recently been proved experimentally that NRF2 controls autophagosome genes, including *ULKl/2*^40^. These data assume potential feedback loops between the main and side switches upon oxidative exposure; however, these connections later must be proven experimentally.

**Fig. 6:**
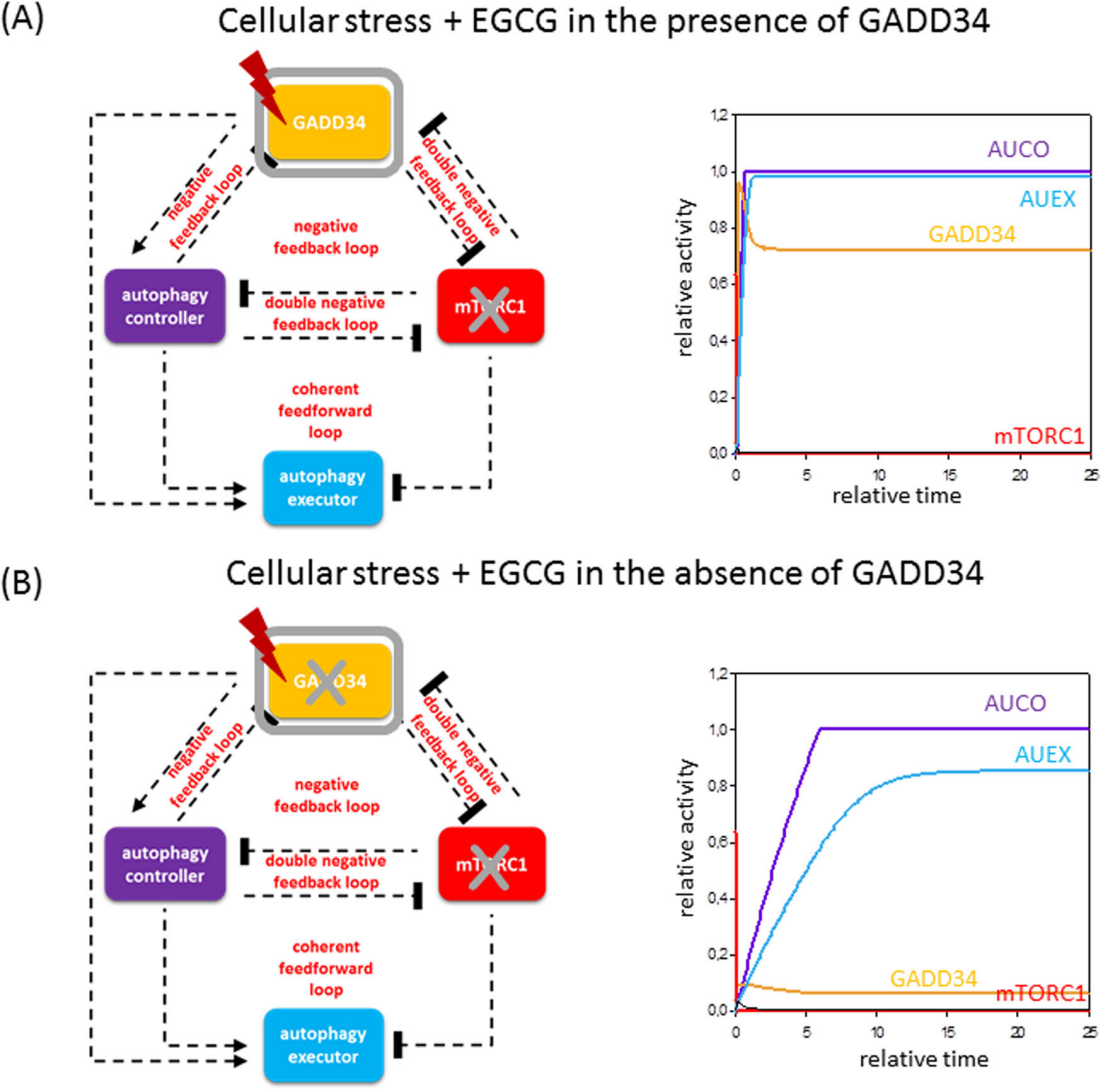
Exploring the mechanism of the lipopolysaccharide (LPS)-induced acute tissue injury with the model system. EGCG ameliorates GADD34 deficiency in LPS-induced acute tissue injury are simulated. The computational simulations determined the EGCG pretreatment (kaai = 50, kimtor = 50) combined with excessive level of cellular stress (stress = 5) in the **A** presence or **B** absence of GADD34 (GADD34-T = 0.1, kaai = 50, kimtor = 50). For details about the codes and software used for simulations, see the Supplementary Information.

To highlight the medical relevance of the presented model, we note that the most commonly occurring complex diseases of the society (i.e., neurodegenerative diseases, metabolic diseases and carcinogenesis) are connected to the malfunction of autophagy. Therefore, we explored the dynamical characteristic of the network controlling autophagy induction. For example, acute lung injury induced by bacterial lipopolysaccharide (LPS) is a common critical illness characterized by inflammatory cytokine expression and cell death, although its molecular mechanism is poorly understood. Ito *et al* has recently revealed that GADD34 attenuates LPS-induced sepsis and acute tissue injury through suppressing macrophage activation, while GADD34 deficiency drastically increased lethality in LPS induction^70^. Interestingly, Wang *et al* has recently shown that addition of EGCG (green tee flavonoid), a natural compound, is able to protect pro-inflammatory cytokine induced injuries in insulin-producing cells through the mitochondrial pathway and therefore ameliorates LPS-induced acute lung injury^71^. Our model supposes that GADD34 is an AUIN (see Figs. 3 and 4). Since EGCG is a well-known AMPK activator (AUIN activator in our model) / mTORC1 inhibitor, question immediately arises, what if EGCG can protect cells in GADD34 deficiency in LPS-induced acute injury? To test this assumption, we simulate EGCG treatment combining with/without GADD34 depletion upon cellular stress (Fig. 6A, B). In case of EGCG treatment (kaai = 50, kimtor =50, stress = 5) the autophagy-dependent survival becomes hyper-active. By depleting GADD34 (AUIN-T = 0.1) during EGCG treatment GADD34 gets decreased, but AUCO (this might be CHOP) remains high resulting in active autophagy-dependent survival. These results need experimental confirmation, but illustrate the way how our systems biology approach can be applied to predict better various treatment and disease scenarios, and the design of the experiments to increase our knowledge on these medically relevant systems. Such better understanding may help us to modulate autophagy-dependent cellular decision with a long-term aim of a possible therapeutical intervention.

Here we introduced a general, controlling network for autophagy that unifies our existing knowledge on how autophagy is regulated in various stress events. We pointed out that the robust response of this control network is essential during cellular stress and the key components, such as switches, could maintain a proper autophagy regulation to enable efficient stress response but inhibit overactivation. Applying this network model concept to other stress events and to disease settings, such as neurodegenerative diseases, cancer and Crohn’s disease, will be a helpful approach to understand the kinetic properties of autophagy regulation in these complex diseases.

## Supplementary information

supplementary material
