# Supplementary material for: Autophagy-dependent survival is controlled with a unique regulatory network upon various cellular stress events
Source: Cell Death Dis. 2021 Mar 23;12(4):309. doi: 10.1038/s41419-021-03599-7 (PMC7988068; doi:10.1038/s41419-021-03599-7)
Supplement: Supplementary file 1 — supplementary material [file 41419_2021_3599_MOESM1_ESM.pdf]

Supplementary Information

"Autophagy-dependent survival is controlled with a unique regulatory network upon various cellular stress events"

Orsolya Kapuy, Marianna Holczer, Margita Márton, Tamás Korcsmáros

I. Mathematical codes for computational simulations

Although the complexity of the cellular system makes really difficult to make mathematical models of a biological regulatory network, with a proper reduction approach a simple wiring diagram can be created containing the key components of the control network (1, 2). Our wiring diagrams are based on the relevant system level feedback loops, while they are independent form can be independent of the identity of molecular players. With this mathematical modelling, the qualitative features of the dynamical system behind autophagy regulation can be easily explored by using both computational simulations and signal-response curves. A biological regulatory network can be translated into a set of ordinary differential equation (ODE) to describe how the concentration/activity of each control element in the network changes with the time (3, 4). A generic differential equation depicting the temporal changes of a regulatory element is composed of two parts: production and consumption terms. In a cellular protein-protein regulatory network the production can be given by protein synthesis (i.e. transcription and translation) and/or an activation (i.e. post-translational modification) term, while the consumption can be given by protein degradation and/or inactivation term. Usually synthesis and degradation reactions are described by mass action kinetics, whereas protein activity can be described either by mass action or Michaelis-Menten kinetics (1, 5-7). Solving a set of non-linear ODEs gives the time evolution of the relative protein concentration/activity (time courses).

The temporal profiles were computed numerically using *XPP-AUT*, which is freely available from http://www.math.pitt.edu/~bard/xpp/xpp.html. All the simulations presented in the text are based on the following XPP codes which contains ODEs. The rate constants (k) have the dimension of min^{-1} and Michaelis constants (J) are dimensionless. The proteins levels/activities are given in arbitrary units (a.u).

The code for a general model simulating time series

```
# a model to simulate autophagy induction with XPP-AUT

# initial conditions
```

```
init AUCO=1.3381e-005, PAUIN=0.0717, AUIN=0.0382, mTOR=0.6370, AUEX=0.0019,
DEATH=0.0044

# differential equations
# AUCO represents the active form of autophagy controller
AUCO' = (kaac + kaac'*AUIN)*(AUCOT-AUCO)/(JAUCO + AUCOT-AUCO) - (kiac +
kiac'*mTOR)*AUCO/(JAUCO + AUCO)

# pAUIN represents the active, pre-form of autophagy inducer
pAUIN' = (kapai + kapai'*stress/(1+stress))*(pAUINT-pAUIN) - (kipai +
kipai'*AUCO + kipai"*mTOR + kipai'"*DEATH)*pAUIN

# AUIN represents the active form of autophagy inducer
AUIN' = kaai*pAUIN*(AUINT-AUIN)/(JAUIN + AUINT-AUIN) - (kiai +
kiai'*mTOR)*AUIN/(JAUIN + AUIN)

# mTOR represents the active form of mTORC1
mTOR' = (kamtor + kamtor'*stress/(1+stress) + kamtor"*DEATH)*(mtoT-mTOR) -
(kimtor + kimtor'*AUIN + kimtor"*AUCO)*mTOR

# AUEX represents the active AUEX genes; when AUEX is active we assume that
autophagy is also active
AUEX' = (kaax + kaax'*AUCO + kaax"*AUIN)*(AUEXT- AUEX)/(JAUEX + AUEXT-
AUEX) - (kiax + kiax'*mTOR + kiax"*DEATH)* AUEX /(JAUEX + AUEX)

# DEATH represents the possible cell death of the cell
DEATH' =(kade + kade'*AUCO + kade"*stress + kade'"*mTOR)*(1-DEATH)/(Jdea +
1-DEATH) - (kide + kide'*AUEX)*DEATH/(Jdea + DEATH)

# parameters
# simulating rapamycin treatment: stress=0.75
# simulating high stress with GADD34 depletion: stress=9, AUIT=0.1
# simulating high stress with CHOP depletion: stress=9, AUCOT=0.1
# simulating high stress with GADD34 over-expression: stress=9, AUIT=2
# simulating high stress with CHOP over-expression: stress=9, AUCOT=1.25
# simulating high stress with KEAP1 depletion: stress=7.5, kaac=5
# simulating EGCG treatment: kaai=50, kimtor=50
# simulating EGCG treatment with high stress: kaai=50, kimtor=50, stress=5
# simulating EGCG treatment with high stress and GADD34 depletion: kaai=50,
kimtor=50, stress=5, AUINT=0.1
p stress=0
p kaac=0.05, kaac'=2, kiac=0.01, kiac'=15, JAUCO=0.001, AUCOT=1
p kapai=0.02, kapai'=1, kipai=0.25, kipai'=20, kipai"=0, kipai'"=2, pAUIT=1
p kaai=7.5, kiai=1.5, kiai'=2, AUINT=1, JAUIN=0.2
p kamtor=0.05, kamtor'=0.03, kamtor"=0.3, kimtor=0.01, kimtor'=0.5,
kimtor"=10, mtoT=1
p kaax=0.075, kaax'=0.15, kaax"=2, kiax=0.1, kiax'=20, kiax"=20, AUEXT=1,
JAUEX=0.2
p kade=0, kade’=0.5, kade”=0.05, kade’”=0.005, kide=0.01, kide’=30,
Jdea=0.1

done
```

The code for a general model calculating balance curves

```
# a model to simulate autophagy induction with XPP-AUT

# initial conditions
init AUCO=0, AUI=0


# differential equations

# AUCO represents the active form of autophagy inducer
AUCO' = (kaac + kaac'*AUIN)*(AUCOT-AUCO)/(JAUCO + AUCOT-AUCO) - (kiac +
kiac'*mTOR)*AUCO/(JAUCO + AUCO)

# AUI represents the active form of autophagy inducer
AUI' = kaai*pAUIN*(AUINT-AUIN)/(JAUI + AUINT-AUIN) - (kiai +
kiai'*mTOR)*AUI/(JAUI + AUIN)
# steady state function

# mTOR represents the active form of mTOR
mTOR = (kamtor + kamtor'*stress/(1+stress) + kamtor"*DEATH)*mtoT/(kamtor +
kamtor'*stress/(1+stress) + kamtor"*DEATH + kimtor + kimtor'*AUIN +
kimtor"*AUCO)

# pAUIN represents the active, pre-form of autophagy inducer
pAUIN = (kapai + kapai'*stress/(1+stress))*pAUINT/(kapai +
kapai'*stress/(1+stress) + kipai + kipai'*AUCO + kipai"*mTOR +
kipai'"*DEATH)

# AUEX represents the active AUEX genes; when AUEX is active we assume that
autophagy is also active
AUEX = AUEXT*GK(kaax + kaax'*AUCO + kaax"*AUIN,kiax + kiax'*mTOR +
kiax"*DEATH,JAUEX,JAUEX)

# DEATH represents the possible cell death of the cell
DEATH = GK(kade + kade'*AUCO + kade"*stress + kade"'*mTOR,kide +
kide'*AUTA,Jdea,Jdea)

# 'Goldbeter-Koshand' function (GK)
GB(arg1,arg2,arg3,arg4) = arg2-arg1+arg2*arg3+arg1*arg4
GK(arg1,arg2,arg3,arg4) =
2*arg1*arg4/(GB(arg1,arg2,arg3,arg4)+sqrt(GB(arg1,arg2,arg3,arg4)^2-
4*(arg2-arg1)*arg1*arg4))

# parameters
# simulating rapamycin treatment: stress=0.75
p stress=0
p kaac=0.05, kaac'=2, kiac=0.01, kiac'=15, jAUCO=0.001, AUCOT=1
p kapai=0.02, kapai'=1, kipai=0.25, kipai'=20, kipai"=0, kipai'"=2,
pAUINT=1
p kaai=7.5, kiai=1.5, kiai'=2, AUINT=1, JAUIN=0.201
p kamtor=0.05, kamtor'=0.03, kamtor"=0.3, kimtor=0.01, kimtor'=0.5,
kimtor"=10, mtoT=1
```

```
p kaax=0.075, kaax'=0.15, kaax"=2, kiax=0.1, kiax'=20, kiax"=20, AUEXT=1,
JAUEX=0.2
p kade=0, kade’=0.5, kade”=0.05, kade’”=0.005, kide=0.01, kide’=30,
Jdea=0.1

done
```

II. Figures

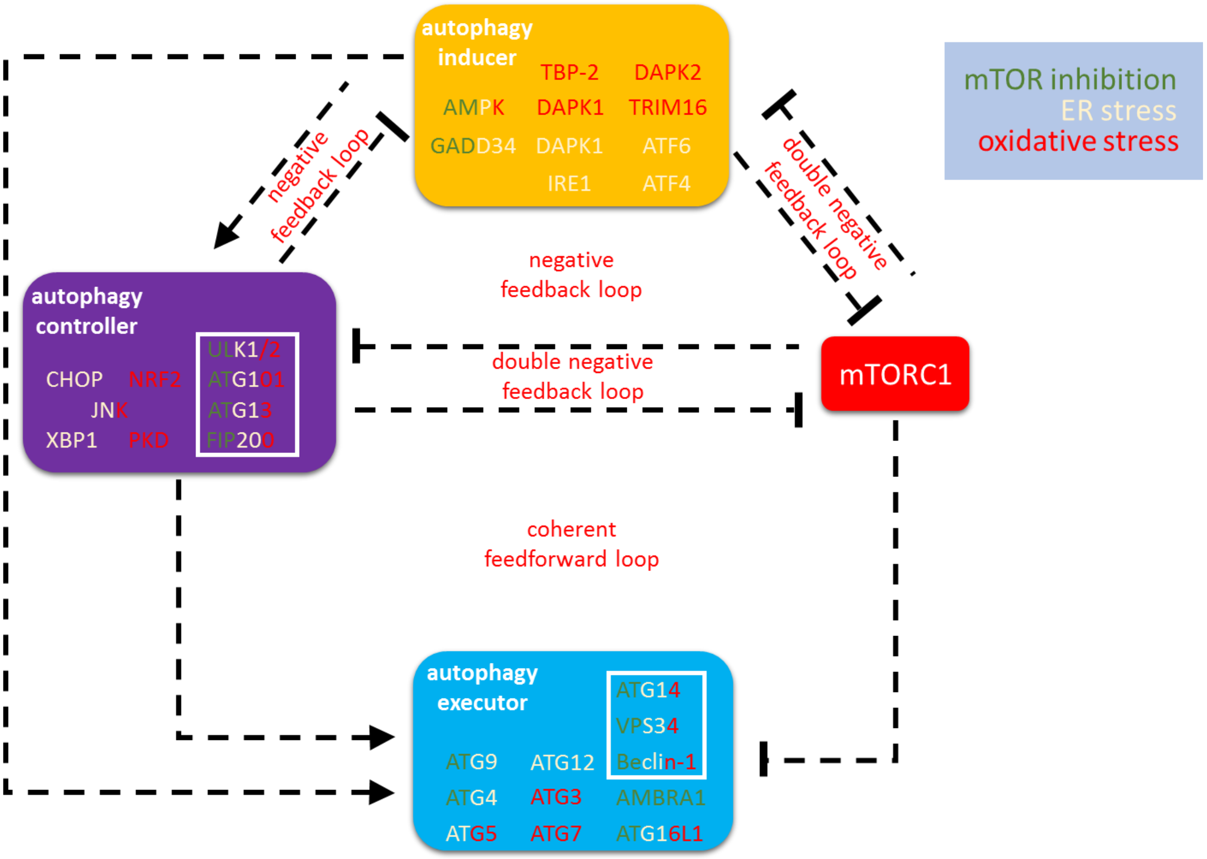

Supplementary Figure 1. The possible regulatory components and their inter-connections in various cellular stress induced autophagy. The autophagy inducer, the autophagy controller, the autophagy executor and mTORC1 are grouped together in isolated orange, purple, red and blue, respectively. Solid arrows represent biochemical reactions, dashed line shows how the molecules can influence each other. Blocked end lines denote inhibition.

III. Tables

	autophagy inducer		autophagy controller		autophagy executors	
	**protein name**	**reference**	**protein name**	**reference**	**protein name**	**reference**
**mTOR inhibition**	AMPK	Kim et al, 2011	ULK1/2	Ganley et al, 2009	AMBRA1	Egan, 2015
		Egan et al, 2011		Hosokawa et al, 2009		Di Bartolomeo et al, 2010
		Leprivier et al, 2020		Jung et al, 2009	ATG14	Wold et al, 2016
		Filomeni et al, 2015		Filomeni et al, 2015	ATG4	Pengo et al, 2017
	GADD34	Ito et al, 2015	FIP200	Ganley et al, 2009	Vps34	Kim et al, 2013
		Uddin et al, 2011		Hosokawa et al, 2009		Russel et al, 2013
		Watanabe et al, 2007		Jung et al, 2009		Egan et al, 2015
		Leprivier et al, 2020		Filomeni et al, 2015	Beclin1	Russel et al, 2013
			ATG13	Ganley et al, 2009		Kim et al, 2013
				Hosokawa et al, 2009		Kim et al, 2013
				Jung et al, 2009	ATG9	Zhou et al, 2017
				Puente et al, 2016		Weerasekara et al, 2014
				Filomeni et al, 2015	ATG16L1	Dooley et al, 2014
			ATG101	Zachari et al, 2017		Gammoh et al, 2013
				Hosokawa et al, 2009		
				Mercer et al, 2009		
**oxidative stress**	AMPK	Kosztelnik et al, 2018	NRF2	Jena et al, 2018	Beclin1	Liu et al, 2015
		Gao, 2019		Filomeni et al, 2015		Lerner et al, 2012
		Shiomi et al, 2014		Navarro-Yepes et al, 2014	VPS34	Lerner et al, 2012
		Filomeni et al, 2015		Medvedev et al, 2016		Lerner et al, 2012
		Navarro-Yepes et al, 2014		Kosztelnik et al, 2018	ATG14	Geering, 2015
		Wang et al, 2011	JNK	Liu et al, 2015	ATG16L	Jena et al, 2018
	TBP-2	Zhou et al, 2016		Lerner et al, 2007	ATG 5	Wang et al, 2015
	DAPK1	Lerner et al, 2012	ULK1/2	Filomeni et al, 2015	ATG7	Wang et al, 2015
		Lerner et al, 2007		Navarro-Yepes et al, 2014		Burgoyne et al, 2018
		Lerner et al, 2012		Jena et al, 2018	ATG3	Burgoyne et al, 2018
	DAPK2	Schlegel et al, 2015	FIP200	Navarro-Yepes et al, 2014	ATG4	Scherz-Shouval et al, 2007
		Geering, 2015		Filomeni et al, 2015		Filomeni et al, 2015
	TRIM16	Jena et al, 2018	ATG13	Navarro-Yepes et al, 2014		Navarro-Yepes et al, 2014
				Filomeni et al, 2015		
			ATG101	Navarro-Yepes et al, 2014		
				Filomeni et al, 2015		
			PKD	Lerner et al, 2012		
				Lerner et al, 2012		
				Lerner et al, 2007		
**ER stress**	AMPK	Kandala et al, 2012	ULK1/2	Song et al, 2018	Beclin-1	Song et al, 2018
		Liu et al, 2018		Lin et al, 2019		Cheng et al, 2014
		Qi et al, 2015	FIP200	Song et al, 2018		Margariti et al, 2013
		Xi et al, 2013		Lin et al, 2019		B'chir et al, 2013
	GADD34	Hyrskyluoto et al, 2012	ATG13	Song et al, 2018		Lin et al, 2019
		Holczer et al, 2016		Lin et al, 2019	VPS34	Song et al, 2018
	DAPK1	Song et al, 2018	ATG101	Song et al, 2018	ATG14	Song et al, 2018
	IRE1	Song et al, 2018		Lin et al, 2019	ATG16L	Song et al, 2018
		Ogata et al, 2006	CHOP	Song et al, 2018		B'chir et al, 2013
		Cheng et al, 2014		B'chir et al, 2013	ATG5	Song et al, 2018
		Margariti et al, 2013		Guo et al, 2014		B'chir et al, 2013
		Lin et al, 2019		Lin et al, 2019		Lin et al, 2019
	ATF4	B'chir et al, 2013	JNK	Song et al, 2018	ATG12	B'chir et al, 2013
		Lin et al, 2019		Ogata et al, 2006		Lin et al, 2019
		Song et al, 2018		Cheng et al, 2014		Song et al, 2018
	ATF6	Song et al, 2018		Lin et al, 2019	ATG9	Lin et al, 2019
		Guo et al, 2014	XBP1	Song et al, 2018	ATG4	Chen et al, 2012
		Lin et al, 2019		Hetz et al, 2009		
				Margariti et al, 2013		
				Guo et al, 2014		

Supplementary Table 1. Collecting data from literature about possible regulatory components of autophagy induction upon various cellular stress.

Connections	References
AMPK -> ULK1/2	Bach et al, 2011
	Egan et al, 2011
	Kim et al, 2011
	Roach, 2011
	Shang et al, 2011
	Urano et al, 2018
	Cardaci et al, 2012
	Kaushal et al, 2019
	Holczer et al, 2018
	Zhao et al, 2016
	Lee et al, 2010
	Behrends et al, 2010
	Tamargo-Gómez et al, 2018
ULK1/2 -I AMPK	Löffler et al, 2011
	Dite et al, 2017
ULK1/2 -I mTORC1	Dunlop et al, 2011
	Jung et al, 2011
AMPK -I mTORC1	Gwinn et al, 2008
	Inoki et al, 2003
	Kaushal et al, 2019
	She et al, 2014
	Alexander et al, 2010
	Zhao et al, 2016
	Meley et al, 2006
	Tamargo-Gómez et al, 2018
mTORC1 -I ULK1/2	Ganley et al, 2009
	Hosokawa et al, 2009
	Kim et al, 2011
	Jung et al, 2009
	Zhao et al, 2016
mTORC1 -I AMPK	Holczer et al, 2019
	Ling et al, 2020
AMPK -> autophagy	Meijer et al, 2015
	Abada et al, 2014
	Xi et al, 2013
	Zhao et al, 2016
	Meley et al, 2006
	Tamargo-Gómez et al, 2018
ULK1/2 -> autophagy	Kim et al, 2011
	Russel et al, 2013
	Urano et al, 2018
	Zhao et al, 2016
	Zachari et al, 2017
mTOR -I autophagy	Nazio et al, 2013
	Yuan et al, 2013
	Settembre et al, 2012
	Martina et al, 2012
	Settembre et al, 2013
	Kim et al, 2015
GADD34 -I mTORC1	Uddin et al, 2011
	Watanabe et al, 2007
	Hyrskyluoto et al, 2012
	Holczer et al, 2016
AMPK -> NRF2	Salminen et al, 2012
	Joo et al, 2016
	Lin et al, 2018
NRF2 -I AMPK	Kosztelnik et al, 2018
GADD34 -> autophagy	Ito et al, 2015
	Uddin et al, 2011
CHOP -> autophagy	B'chir et al, 2013
	Rouschop et al, 2010
IRE1 -> autophagy	Song et al, 2018
	Ogata et al, 2006

mTOR inhibition (starvation, rapamycin treatment, etc.)
oxidative stress
ER stress
oxidative stress + mTOR inhibition
ER stress + mTOR inhibition
none of them

Supplementary Table 2. Collecting data from literature about the proved regulatory connections during cellular stress induced autophagy regulation.

IV. Introducing the theoretical analysis of phosphorylation site search on NRF2

The potential Ser and Thr phosphorylation sites of ULK1/2 were identified on NRF2 sequence by Group-based Prediction System 5.0 (http://gps.biocuckoo.cn/). The sequence of NRF2 was downloaded from UniProt (https://www.uniprot.org/). The threshold was high under prediction. Table 3. includes the position of Ser and Thr amino acids, the catalytic subunits of ULK1/2, the peptid sequence in the near of Ser and Thr amino acids and the score values. The score value is calculated by GPS algorithm to evaluate the potential of phosphorylation. The higher the value, the more potential the residue is phosphorylated (http://gps.biocuckoo.cn/).

The phosphorylation sites were verified by NetPhos 3.1 (http://www.cbs.dtu.dk/services/NetPhos). Those phosphorylation sites were collected and checked where the phosphorylation kinase was unknown (see the NetPhos column in the Table 3.). The score above 0.500 indicates positive predictions.

The phosphorylation sites were also searched with the help of PhosphositePlus (https://www.phosphosite.org/homeAction.action). In this database, the consensus phosphorylation motif of ULK1 is used, which is created with the help of several well-known phosphorylated sequences of ULK1 substrates. The same amino acids within the identified potential ULK1 phosphorylation sequences were marked with red colour (see the Peptid column of Table 3.).

Detailed description the legend of Supplementary Table 3.

ULK1/2 phosphorylation sites on NRF2					NetPhos
**Position**	**Code**	**Enzyme**	**Peptide**	**Score**	**Score - kinase (unknown)**
the position number # of S/T P'ion site	Ser (S) or Thr (T) P'ion site	name of the kinase	consensus P'ion sequence with red letters	evaluate the potential of P'ion site	evaluate the potential of P'ion site

Consensus ULK1-dependent phosphorylation sites

PhosphoSite Plus shows the preferred Ser and Thr phosphorylation sites of ULK1 kinase:
https://www.phosphosite.org/proteinAction.action?id=796&showAllSites=true

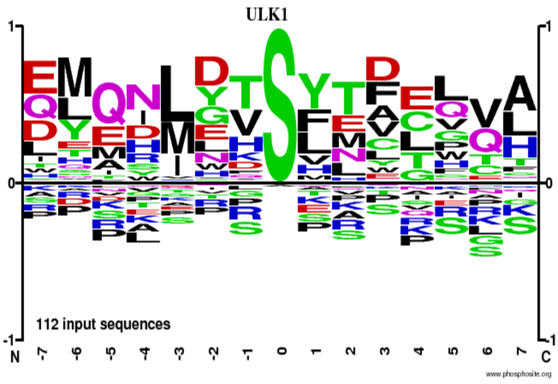

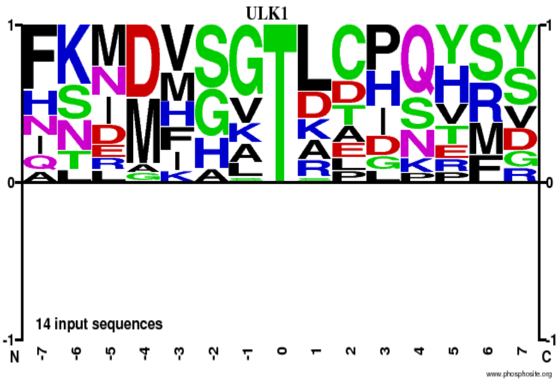

The preferred Ser and Thr phosphorylation sites of ULK2 kinase is not known yet, however the protein sequence identity is 78% within the kinase domains of ULK1 and ULK2 (8).

Table 3. *ULK1/2 phosphorylation sites on different isoforms of NRF2*

ULK1/2 phosphorylation sites on NRF2 Isoform 1					NetPhos
**Position**	**Code**	**Enzyme**	**Peptide**	**Score**	**Score - kinase (unknown)**
351	S	ULK1	SGISLNTSPSVASPE	38.529	0.981 unsp
410	S	ULK1	MVQPLSPSQGQSTHV	42.467	
231	S	ULK2	DNYHFYSSIPSMEKE	4.744	
320	S	ULK2	LNGPIDVSDLSLCKA	4.537	
414	S	ULK2	LSPSQGQSTHVHDAQ	5.696	0.638 unsp
447	S	ULK2	PFTKDKHSSRLEAHL	4.241	0.992 unsp
**ULK1/2 phosphorylation sites on NRF2 Isoform 2**					**NetPhos**
**Position**	**Code**	**Enzyme**	**Peptide**	**Score**	**Score - kinase (unknown)**
335	S	ULK1	SGISLNTSPSVASPE	38.529	0.981 unsp
394	S	ULK1	MVQPLSPSQGQSTHV	42.467	
17	S	ULK2	QDIDLGVSREVFDFS	4.638	0.971 unsp
215	S	ULK2	DNYHFYSSIPSMEKE	4.744	
304	S	ULK2	LNGPIDVSDLSLCKA	4.537	
398	S	ULK2	LSPSQGQSTHVHDAQ	5.696	0.638 unsp
431	S	ULK2	PFTKDKHSSRLEAHL	4.241	0.992 unsp
**ULK1/2 phosphorylation sites on NRF2 Isoform 3**					**NetPhos**
**Position**	**Code**	**Enzyme**	**Peptide**	**Score**	**Score - kinase (unknown)**
328	S	ULK1	SGISLNTSPSVASPE	38.529	0.981 unsp
387	S	ULK1	MVQPLSPSQGQSTHV	42.467	
17	S	ULK2	QDIDLGVSREVFDFS	4.638	0.971 unsp
208	S	ULK2	DNYHFYSSIPSMEKE	4.744	
297	S	ULK2	LNGPIDVSDLSLCKA	4.537	
391	S	ULK2	LSPSQGQSTHVHDAQ	5.696	0.638 unsp
424	S	ULK2	PFTKDKHSSRLEAHL	4.241	0.992 unsp

V. References

1. Tyson JJ, Chen KC, Novak B. Sniffers, buzzers, toggles and blinkers: dynamics of regulatory and signaling pathways in the cell. Curr Opin Cell Biol. 2003;15(2):221-31.
2. Tyson JJ, Novak B. Functional motifs in biochemical reaction networks. Annu Rev Phys Chem. 2010;61:219-40.
3. Tyson JJ, Chen K, Novak B. Network dynamics and cell physiology. Nature Rev Mol Cell Biol. 2001;2:908-16.
4. Strogatz SH. Nonlinear dynamics and Chaos : with applications to physics, biology, chemistry, and engineering. Reading, Mass.: Addison-Wesley Pub.; 1994. xi, 498 p. p.
5. Segel IH. Enzyme kinetics behavior and analysis of rapid equilibrium and steady state enzyme systems: Wiley; 1975.
6. Goldbeter A, Koshland DE, Jr. An amplified sensitivity arising from covalent modification in biological systems. Proc Natl Acad Sci U S A. 1981;78(11):6840-4.
7. Tyson JJ, Csikasz-Nagy A, Novak B. The dynamics of cell cycle regulation. BioEssays. 2002;24:1095-109.
8. Zachari M, Ganley IG. The mammalian ULK1 complex and autophagy initiation. Essays Biochem. 2017;61(6):585-96.
9. Abada A, Elazar Z. Getting ready for building: signaling and autophagosome biogenesis. EMBO Rep. 2014;15(8):839-52.
10. Alexander A, Cai SL, Kim J, Nanez A, Sahin M, MacLean KH, et al. ATM signals to TSC2 in the cytoplasm to regulate mTORC1 in response to ROS. Proc Natl Acad Sci U S A. 2010;107(9):4153-8.
11. B'Chir W, Maurin AC, Carraro V, Averous J, Jousse C, Muranishi Y, et al. The eIF2alpha/ATF4 pathway is essential for stress-induced autophagy gene expression. Nucleic Acids Res. 2013;41(16):7683-99.
12. Bach M, Larance M, James DE, Ramm G. The serine/threonine kinase ULK1 is a target of multiple phosphorylation events. Biochem J. 2011;440(2):283-91.
13. Behrends C, Sowa ME, Gygi SP, Harper JW. Network organization of the human autophagy system. Nature. 2010;466(7302):68-76.
14. Burgoyne JR. Oxidative stress impairs autophagy through oxidation of ATG3 and ATG7. Autophagy. 2018;14(6):1092-3.
15. Cardaci S, Filomeni G, Ciriolo MR. Redox implications of AMPK-mediated signal transduction beyond energetic clues. J Cell Sci. 2012;125(Pt 9):2115-25.
16. Cheng X, Liu H, Jiang CC, Fang L, Chen C, Zhang XD, et al. Connecting endoplasmic reticulum stress to autophagy through IRE1/JNK/beclin-1 in breast cancer cells. Int J Mol Med. 2014;34(3):772-81.
17. Di Bartolomeo S, Corazzari M, Nazio F, Oliverio S, Lisi G, Antonioli M, et al. The dynamic interaction of AMBRA1 with the dynein motor complex regulates mammalian autophagy. J Cell Biol. 2010;191(1):155-68.
18. Dite TA, Ling NXY, Scott JW, Hoque A, Galic S, Parker BL, et al. The autophagy initiator ULK1 sensitizes AMPK to allosteric drugs. Nat Commun. 2017;8(1):571.
19. Dooley HC, Razi M, Polson HE, Girardin SE, Wilson MI, Tooze SA. WIPI2 links LC3 conjugation with PI3P, autophagosome formation, and pathogen clearance by recruiting Atg12-5-16L1. Mol Cell. 2014;55(2):238-52.
20. Dunlop EA, Hunt DK, Acosta-Jaquez HA, Fingar DC, Tee AR. ULK1 inhibits mTORC1 signaling, promotes multisite Raptor phosphorylation and hinders substrate binding. Autophagy. 2011;7(7):737-47.
21. Egan DF, Chun MG, Vamos M, Zou H, Rong J, Miller CJ, et al. Small Molecule Inhibition of the Autophagy Kinase ULK1 and Identification of ULK1 Substrates. Mol Cell. 2015;59(2):285-97.
22. Egan DF, Shackelford DB, Mihaylova MM, Gelino S, Kohnz RA, Mair W, et al. Phosphorylation of ULK1 (hATG1) by AMP-activated protein kinase connects energy sensing to mitophagy. Science. 2011;331(6016):456-61.
23. Eisenberg-Lerner A, Kimchi A. DAP kinase regulates JNK signaling by binding and activating protein kinase D under oxidative stress. Cell Death Differ. 2007;14(11):1908-15.
24. Eisenberg-Lerner A, Kimchi A. PKD is a kinase of Vps34 that mediates ROS-induced autophagy downstream of DAPk. Cell Death Differ. 2012;19(5):788-97.
25. Eisenberg-Lerner A, Kimchi A. PKD at the crossroads of necrosis and autophagy. Autophagy. 2012;8(3):433-4.
26. Filomeni G, De Zio D, Cecconi F. Oxidative stress and autophagy: the clash between damage and metabolic needs. Cell Death Differ. 2015;22(3):377-88.
27. Gammoh N, Florey O, Overholtzer M, Jiang X. Interaction between FIP200 and ATG16L1 distinguishes ULK1 complex-dependent and -independent autophagy. Nat Struct Mol Biol. 2013;20(2):144-9.

28. Ganley IG, Lam du H, Wang J, Ding X, Chen S, Jiang X. ULK1.ATG13.FIP200 complex mediates mTOR signaling and is essential for autophagy. J Biol Chem. 2009;284(18):12297-305.
29. Gao Q. Oxidative Stress and Autophagy. Adv Exp Med Biol. 2019;1206:179-98.
30. Geering B. Death-associated protein kinase 2: Regulator of apoptosis, autophagy and inflammation. Int J Biochem Cell Biol. 2015;65:151-4.
31. Guo FJ, Xiong Z, Lu X, Ye M, Han X, Jiang R. ATF6 upregulates XBP1S and inhibits ER stress-mediated apoptosis in osteoarthritis cartilage. Cell Signal. 2014;26(2):332-42.
32. Gwinn DM, Shackelford DB, Egan DF, Mihaylova MM, Mery A, Vasquez DS, et al. AMPK phosphorylation of raptor mediates a metabolic checkpoint. Mol Cell. 2008;30(2):214-26.
33. Hetz C, Thielen P, Matus S, Nassif M, Court F, Kiffin R, et al. XBP-1 deficiency in the nervous system protects against amyotrophic lateral sclerosis by increasing autophagy. Genes Dev. 2009;23(19):2294-306.
34. Holczer M, Banhegyi G, Kapuy O. GADD34 Keeps the mTOR Pathway Inactivated in Endoplasmic Reticulum Stress Related Autophagy. PLoS One. 2016;11(12):e0168359.
35. Holczer M, Besze B, Zambo V, Csala M, Banhegyi G, Kapuy O. Epigallocatechin-3-Gallate (EGCG) Promotes Autophagy-Dependent Survival via Influencing the Balance of mTOR-AMPK Pathways upon Endoplasmic Reticulum Stress. Oxid Med Cell Longev. 2018;2018:6721530.
36. Holczer M, Hajdu B, Lorincz T, Szarka A, Banhegyi G, Kapuy O. A Double Negative Feedback Loop between mTORC1 and AMPK Kinases Guarantees Precise Autophagy Induction upon Cellular Stress. Int J Mol Sci. 2019;20(22).
37. Hosokawa N, Hara T, Kaizuka T, Kishi C, Takamura A, Miura Y, et al. Nutrient-dependent mTORC1 association with the ULK1-Atg13-FIP200 complex required for autophagy. Mol Biol Cell. 2009;20(7):1981-91.
38. Hosokawa N, Sasaki T, Iemura S, Natsume T, Hara T, Mizushima N. Atg101, a novel mammalian autophagy protein interacting with Atg13. Autophagy. 2009;5(7):973-9.
39. Hyrskyluoto A, Reijonen S, Kivinen J, Lindholm D, Korhonen L. GADD34 mediates cytoprotective autophagy in mutant huntingtin expressing cells via the mTOR pathway. Exp Cell Res. 2012;318(1):33-42.
40. Inoki K, Zhu T, Guan KL. TSC2 mediates cellular energy response to control cell growth and survival. Cell. 2003;115(5):577-90.
41. Ito S, Tanaka Y, Oshino R, Aiba K, Thanasegaran S, Nishio N, et al. GADD34 inhibits activation-induced apoptosis of macrophages through enhancement of autophagy. Sci Rep. 2015;5:8327.
42. Jena KK, Kolapalli SP, Mehto S, Nath P, Das B, Sahoo PK, et al. TRIM16 controls assembly and degradation of protein aggregates by modulating the p62-NRF2 axis and autophagy. EMBO J. 2018;37(18).
43. Joo MS, Kim WD, Lee KY, Kim JH, Koo JH, Kim SG. AMPK Facilitates Nuclear Accumulation of Nrf2 by Phosphorylating at Serine 550. Mol Cell Biol. 2016;36(14):1931-42.
44. Jung CH, Jun CB, Ro SH, Kim YM, Otto NM, Cao J, et al. ULK-Atg13-FIP200 complexes mediate mTOR signaling to the autophagy machinery. Mol Biol Cell. 2009;20(7):1992-2003.
45. Jung CH, Seo M, Otto NM, Kim DH. ULK1 inhibits the kinase activity of mTORC1 and cell proliferation. Autophagy. 2011;7(10):1212-21.
46. Kandala PK, Srivastava SK. Regulation of macroautophagy in ovarian cancer cells in vitro and in vivo by controlling glucose regulatory protein 78 and AMPK. Oncotarget. 2012;3(4):435-49.
47. Kaushal GP, Chandrashekar K, Juncos LA. Molecular Interactions Between Reactive Oxygen Species and Autophagy in Kidney Disease. Int J Mol Sci. 2019;20(15).
48. Kim J, Guan KL. AMPK connects energy stress to PIK3C3/VPS34 regulation. Autophagy. 2013;9(7):1110-1.
49. Kim J, Kim YC, Fang C, Russell RC, Kim JH, Fan W, et al. Differential regulation of distinct Vps34 complexes by AMPK in nutrient stress and autophagy. Cell. 2013;152(1-2):290-303.
50. Kim J, Kundu M, Viollet B, Guan KL. AMPK and mTOR regulate autophagy through direct phosphorylation of Ulk1. Nat Cell Biol. 2011;13(2):132-41.
51. Kim YC, Guan KL. mTOR: a pharmacologic target for autophagy regulation. J Clin Invest. 2015;125(1):25-32.
52. Kosztelnik M, Kurucz A, Papp D, Jones E, Sigmond T, Barna J, et al. Suppression of AMPK/aak-2 by NRF2/SKN-1 down-regulates autophagy during prolonged oxidative stress. FASEB J. 2019;33(2):2372-87.
53. Lee JW, Park S, Takahashi Y, Wang HG. The association of AMPK with ULK1 regulates autophagy. PLoS One. 2010;5(11):e15394.
54. Leprivier G, Rotblat B. How does mTOR sense glucose starvation? AMPK is the usual suspect. Cell Death Discov. 2020;6:27.
55. Lin Y, Jiang M, Chen W, Zhao T, Wei Y. Cancer and ER stress: Mutual crosstalk between autophagy, oxidative stress and inflammatory response. Biomed Pharmacother. 2019;118:109249.

56. Ling NXY, Kaczmarek A, Hoque A, Davie E, Ngoei KRW, Morrison KR, et al. mTORC1 directly inhibits AMPK to promote cell proliferation under nutrient stress. Nat Metab. 2020;2(1):41-9.
57. Liu GY, Jiang XX, Zhu X, He WY, Kuang YL, Ren K, et al. ROS activates JNK-mediated autophagy to counteract apoptosis in mouse mesenchymal stem cells in vitro. Acta Pharmacol Sin. 2015;36(12):1473-9.
58. Liu JQ, Zhang L, Yao J, Yao S, Yuan T. AMPK alleviates endoplasmic reticulum stress by inducing the ER-chaperone ORP150 via FOXO1 to protect human bronchial cells from apoptosis. Biochem Biophys Res Commun. 2018;497(2):564-70.
59. Loffler AS, Alers S, Dieterle AM, Keppeler H, Franz-Wachtel M, Kundu M, et al. Ulk1-mediated phosphorylation of AMPK constitutes a negative regulatory feedback loop. Autophagy. 2011;7(7):696-706.
60. Margariti A, Li H, Chen T, Martin D, Vizcay-Barrena G, Alam S, et al. XBP1 mRNA splicing triggers an autophagic response in endothelial cells through BECLIN-1 transcriptional activation. J Biol Chem. 2013;288(2):859-72.
61. Martina JA, Chen Y, Gucek M, Puertollano R. MTORC1 functions as a transcriptional regulator of autophagy by preventing nuclear transport of TFEB. Autophagy. 2012;8(6):903-14.
62. Medvedev R, Hildt E, Ploen D. Look who's talking-the crosstalk between oxidative stress and autophagy supports exosomal-dependent release of HCV particles. Cell Biol Toxicol. 2017;33(3):211-31.
63. Meijer AJ, Lorin S, Blommaart EF, Codogno P. Regulation of autophagy by amino acids and MTOR-dependent signal transduction. Amino Acids. 2015;47(10):2037-63.
64. Meley D, Bauvy C, Houben-Weerts JH, Dubbelhuis PF, Helmond MT, Codogno P, et al. AMP-activated protein kinase and the regulation of autophagic proteolysis. J Biol Chem. 2006;281(46):34870-9.
65. Mercer CA, Kaliappan A, Dennis PB. A novel, human Atg13 binding protein, Atg101, interacts with ULK1 and is essential for macroautophagy. Autophagy. 2009;5(5):649-62.
66. Navarro-Yepes J, Burns M, Anandhan A, Khalimonchuk O, del Razo LM, Quintanilla-Vega B, et al. Oxidative stress, redox signaling, and autophagy: cell death versus survival. Antioxid Redox Signal. 2014;21(1):66-85.
67. Nazio F, Strappazzon F, Antonioli M, Bielli P, Cianfanelli V, Bordi M, et al. mTOR inhibits autophagy by controlling ULK1 ubiquitylation, self-association and function through AMBRA1 and TRAF6. Nat Cell Biol. 2013;15(4):406-16.
68. Ogata M, Hino S, Saito A, Morikawa K, Kondo S, Kanemoto S, et al. Autophagy is activated for cell survival after endoplasmic reticulum stress. Mol Cell Biol. 2006;26(24):9220-31.
69. Pengo N, Agrotis A, Prak K, Jones J, Ketteler R. A reversible phospho-switch mediated by ULK1 regulates the activity of autophagy protease ATG4B. Nat Commun. 2017;8(1):294.
70. Qi D, Young LH. AMPK: energy sensor and survival mechanism in the ischemic heart. Trends Endocrinol Metab. 2015;26(8):422-9.
71. Roach PJ. AMPK -> ULK1 -> autophagy. Mol Cell Biol. 2011;31(15):3082-4.
72. Rouschop KM, van den Beucken T, Dubois L, Niessen H, Bussink J, Savelkouls K, et al. The unfolded protein response protects human tumor cells during hypoxia through regulation of the autophagy genes MAP1LC3B and ATG5. J Clin Invest. 2010;120(1):127-41.
73. Russell RC, Tian Y, Yuan H, Park HW, Chang YY, Kim J, et al. ULK1 induces autophagy by phosphorylating Beclin-1 and activating VPS34 lipid kinase. Nat Cell Biol. 2013;15(7):741-50.
74. Salminen A, Kaarniranta K. AMP-activated protein kinase (AMPK) controls the aging process via an integrated signaling network. Ageing Res Rev. 2012;11(2):230-41.
75. Scherz-Shouval R, Shvets E, Fass E, Shorer H, Gil L, Elazar Z. Reactive oxygen species are essential for autophagy and specifically regulate the activity of Atg4. EMBO J. 2007;26(7):1749-60.
76. Schlegel CR, Georgiou ML, Misterek MB, Stocker S, Chater ER, Munro CE, et al. DAPK2 regulates oxidative stress in cancer cells by preserving mitochondrial function. Cell Death Dis. 2015;6:e1671.
77. Settembre C, Fraldi A, Medina DL, Ballabio A. Signals from the lysosome: a control centre for cellular clearance and energy metabolism. Nat Rev Mol Cell Biol. 2013;14(5):283-96.
78. Settembre C, Zoncu R, Medina DL, Vetrini F, Erdin S, Erdin S, et al. A lysosome-to-nucleus signalling mechanism senses and regulates the lysosome via mTOR and TFEB. EMBO J. 2012;31(5):1095-108.
79. Shang L, Chen S, Du F, Li S, Zhao L, Wang X. Nutrient starvation elicits an acute autophagic response mediated by Ulk1 dephosphorylation and its subsequent dissociation from AMPK. Proc Natl Acad Sci U S A. 2011;108(12):4788-93.
80. She C, Zhu LQ, Zhen YF, Wang XD, Dong QR. Activation of AMPK protects against hydrogen peroxide-induced osteoblast apoptosis through autophagy induction and NADPH maintenance: new implications for osteonecrosis treatment? Cell Signal. 2014;26(1):1-8.

81. Shiomi M, Miyamae M, Takemura G, Kaneda K, Inamura Y, Onishi A, et al. Sevoflurane induces cardioprotection through reactive oxygen species-mediated upregulation of autophagy in isolated guinea pig hearts. J Anesth. 2014;28(4):593-600.
82. Song S, Tan J, Miao Y, Zhang Q. Crosstalk of ER stress-mediated autophagy and ER-phagy: Involvement of UPR and the core autophagy machinery. J Cell Physiol. 2018;233(5):3867-74.
83. Tamargo-Gomez I, Marino G. AMPK: Regulation of Metabolic Dynamics in the Context of Autophagy. Int J Mol Sci. 2018;19(12).
84. Uddin MN, Ito S, Nishio N, Suganya T, Isobe K. Gadd34 induces autophagy through the suppression of the mTOR pathway during starvation. Biochem Biophys Res Commun. 2011;407(4):692-8.
85. Urano Y, Mori C, Fuji A, Konno K, Yamamoto T, Yashirogi S, et al. 6-Hydroxydopamine induces secretion of PARK7/DJ-1 via autophagy-based unconventional secretory pathway. Autophagy. 2018;14(11):1943-58.
86. Wang Q, Liang B, Shirwany NA, Zou MH. 2-Deoxy-D-glucose treatment of endothelial cells induces autophagy by reactive oxygen species-mediated activation of the AMP-activated protein kinase. PLoS One. 2011;6(2):e17234.
87. Wang T, Wang Q, Song R, Zhang Y, Zhang K, Yuan Y, et al. Autophagy Plays a Cytoprotective Role During Cadmium-Induced Oxidative Damage in Primary Neuronal Cultures. Biol Trace Elem Res. 2015;168(2):481-9.
88. Watanabe R, Tambe Y, Inoue H, Isono T, Haneda M, Isobe K, et al. GADD34 inhibits mammalian target of rapamycin signaling via tuberous sclerosis complex and controls cell survival under bioenergetic stress. Int J Mol Med. 2007;19(3):475-83.
89. Weerasekara VK, Panek DJ, Broadbent DG, Mortenson JB, Mathis AD, Logan GN, et al. Metabolic-stress-induced rearrangement of the 14-3-3zeta interactome promotes autophagy via a ULK1- and AMPK-regulated 14-3-3zeta interaction with phosphorylated Atg9. Mol Cell Biol. 2014;34(24):4379-88.
90. Wold MS, Lim J, Lachance V, Deng Z, Yue Z. ULK1-mediated phosphorylation of ATG14 promotes autophagy and is impaired in Huntington's disease models. Mol Neurodegener. 2016;11(1):76.
91. Xi H, Barredo JC, Merchan JR, Lampidis TJ. Endoplasmic reticulum stress induced by 2-deoxyglucose but not glucose starvation activates AMPK through CaMKKbeta leading to autophagy. Biochem Pharmacol. 2013;85(10):1463-77.
92. Yuan HX, Russell RC, Guan KL. Regulation of PIK3C3/VPS34 complexes by MTOR in nutrient stress-induced autophagy. Autophagy. 2013;9(12):1983-95.
93. Zachari M, Ganley IG. The mammalian ULK1 complex and autophagy initiation. Essays Biochem. 2017;61(6):585-96.
94. Zhao F, Huang W, Zhang Z, Mao L, Han Y, Yan J, et al. Triptolide induces protective autophagy through activation of the CaMKKbeta-AMPK signaling pathway in prostate cancer cells. Oncotarget. 2016;7(5):5366-82.
95. Zhou C, Ma K, Gao R, Mu C, Chen L, Liu Q, et al. Regulation of mATG9 trafficking by Src- and ULK1-mediated phosphorylation in basal and starvation-induced autophagy. Cell Res. 2017;27(2):184-201.
96. Zhou J, Yao K, Zhang Y, Chen G, Lai K, Yin H, et al. Thioredoxin Binding Protein-2 Regulates Autophagy of Human Lens Epithelial Cells under Oxidative Stress via Inhibition of Akt Phosphorylation. Oxid Med Cell Longev. 2016;2016:4856431.
